# Nitenpyram seed treatment effectively controls against the mirid bug *Apolygus lucorum* in cotton seedlings

**DOI:** 10.1038/s41598-017-09251-9

**Published:** 2017-08-17

**Authors:** Zhengqun Zhang, Yao Wang, Yunhe Zhao, Beixing Li, Jin Lin, Xuefeng Zhang, Feng Liu, Wei Mu

**Affiliations:** 1 College of Plant Protection, Shandong Agricultural University, 61 Daizong Street, Tai’an, 271018 China; 2 College of Horticulture Science and Engineering, Shandong Agricultural University, 61 Daizong Street, Tai’an, 271018 China

## Abstract

The mirid bug *Apolygus lucorum* (Meyer-Dür) has become a major pest in cotton fields and has led to significant yield losses due to the widespread use of transgenic Bt cotton in China. Eight neonicotinoid seed treatments were investigated to determine their effects on the management of *A. lucorum* in cotton fields. All neonicotinoid seed treatments reduced the cotton damage caused by *A. lucorum*, and nitenpyram at the rate of 4 g/kg seed showed the most favorable efficacy in suppressing *A. lucorum* populations throughout the cotton seedling stage. The neonicotinoid seed treatments had no effect on the emergence rate of cotton seeds. Although the neonicotinoid seed treatments were not significantly different from the spray treatments in the cotton yield, the seed treatments reduced the need for three pesticide applications and showed a tremendous advantage in labor costs throughout the cotton seedling stage. Overall, the neonicotinoid seed treatments, particularly the nitenpyram seed treatment, can provide effective protection and should play an important role in the management of early season *A. lucorum* in Bt cotton fields.

## Introduction

The mirid bug *Apolygus lucorum* (Meyer-Dür) (Hemiptera: Miridae) is an economically important insect pest of Bt cotton in Northern China^1^. This polyphagous pest also has a wide range of host plants, including many arable crops, vegetables, stone fruits, ornamentals, and pasture plants^2, 3^. *A*. *lucorum* adults and nymphs feeding on cotton plants result in bud blast, flower abortion, and missing or shrunken squares and bolls. These abnormalities arise primarily from the activity of polygalacturonase in the salivary glands of this pest^4^. *Apolygus lucorum* causes extremely large cotton yield losses of up to 20–30% every year^5^.

The main control measures for *A. lucorum* in cotton fields in China include chemical control, cultural control (e.g., intercropping with trap crops), physical control (e.g., light traps and sticky traps), and biological control (e.g., releasing parasitic wasps and conserving and utilizing natural enemies)^2^. However, due to their high mobility, cryptic damage, and broad host range, various strategies for the management of *A. lucorum* have not reached the optimal effect^6^. Currently, cotton farmers rely primarily on foliar spraying application of insecticides, including organophosphates, pyrethroids, and neonicotinoids, to manage *A. lucorum* in cotton fields due to its rapid action and high efficiency. However, because of the short residual effects of sprayed insecticides and the resistance developed against insecticides that are commonly used, cotton farmers have to spray foliar insecticides approximately 2–3 times to manage *A. lucorum* during the seedling stage, which increases pesticides use and labor output^6, 7^. Additionally, the best timing for foliar applications in the field is usually difficult to determine based on the probability of an outbreak of pests, and the unavoidable delay in applying insecticides can cause production loss^8^. Frequently applied insecticides also kill natural enemies in cotton fields and weaken their biocontrol services^9^. In addition to the direct mortality induced by insecticides, their sub-lethal effects on the physiology and behavior of beneficial arthropods interact with the life-history traits involved in reproduction (i.e., foraging, fecundity, sexual communication, and sex ratio) and have an impact on beneficial arthropod communities^10^. Seed treatments have represented an important measure in integrated pest management systems because these treatments directly protect crops against seed and root feeders and early season foliar pests and decrease applicator exposure and the amount of active ingredient used^11, 12^. Seed treatments with systemic insecticides could provide a longer-term protection and could have fewer side effects on natural enemies than spraying applications^13^. Neonicotinoid insecticides are nicotinic acetylcholine receptor agonists that exhibit excellent biological activity against a wide range of foliar and soil insect pests, including aphids, whiteflies, leafhoppers, beetles, and rootworms, on various agricultural crop plants through contact or ingestion^14^. Currently, neonicotinoids are among the most widely used class of insecticides worldwide^15^. Moreover, neonicotinoid insecticides have excellent systemic characteristics and are suitable for use as seed treatments for the management of sucking insect pests and certain chewing species that affect seedling stage crops^14, 16, 17^. Currently, approximately 60% of all neonicotinoid applications are soil/seed treatments^13^. Previous studies have shown that neonicotinoid seed treatments provide early season seedling protection against a range of sucking pests, such as *Aphis gossypii*, *Bemisia tabaci*, and *Amrasca devastans*, in cotton fields^11, 18, 19^.

The objective of this study was to evaluate the efficacy of eight neonicotinoids used as seed treatments for the management of *A. lucorum* infestations at the seedling stage of cotton. The data can be used to select efficient neonicotinoids as seed treatments suitable to improve the control of *A. lucorum* in cotton fields in China.

## Results

### Emergence rate of cotton seeds with different treatments

The emergence rate of cotton seeds with neonicotinoid treatments in the laboratory were all approximately 90–93% (*F*_8,53_ = 0.751, *P* = 0.647), and no significant differences were found among the different treatments in the field (2013: *F*_8,35_ = 0.327, *P* = 0.948; 2014: *F*_8,35_ = 0.354, *P* = 0.936; 2015: *F*_8,35_ = 0.280, *P* = 0.967) (Table 1).

**Table 1:** Effects of the neonicotinoid seed treatments on the germination of seeds in the laboratory and the emergence of seedlings in the field.

Insecticide	Germination rate (%)	No. of cotton seedlings in each plot		
		2013	2014	2015
Imidacloprid	92.83 ± 1.08 a	655.0 ± 16.4 a	620.8 ± 13.1 a	587.5 ± 11.0 a
Thiamethoxam	92.17 ± 0.91 a	663.3 ± 14.4 a	633.3 ± 12.6 a	597.5 ± 9.3 a
Clothianidin	90.67 ± 0.88 a	654.5 ± 10.4 a	629.5 ± 7.9 a	600.0 ± 15.3 a
Nitenpyram	92.67 ± 0.95 a	663.3 ± 16.0 a	635.0 ± 11.4 a	605.8 ± 11.4 a
Dinotefuran	90.83 ± 1.22 a	647.0 ± 16.4 a	638.5 ± 16.7 a	605.0 ± 11.5 a
Acetamiprid	92.17 ± 1.01 a	639.0 ± 14.8 a	618.3 ± 11.4 a	597.5 ± 11.8 a
Sulfoxaflor	91.17 ± 0.83 a	645.8 ± 15.7 a	625.3 ± 10.6 a	595.3 ± 9.4 a
Thiacloprid	89.67 ± 1.91 a	642.8 ± 15.8 a	630.0 ± 13.2 a	597.0 ± 11.1 a
Untreated control	92.17 ± 1.72 a	658.5 ± 18.4 a	638.0 ± 10.7 a	606.0 ± 9.8 a

Values within columns represent the means ± SEM.

Different letters indicate significant differences among the different treatments (Tukey’s HSD test, *P* < 0.05).

### Effect of neonicotinoid seed treatments on an *A. lucorum* population

**Figure 1:**
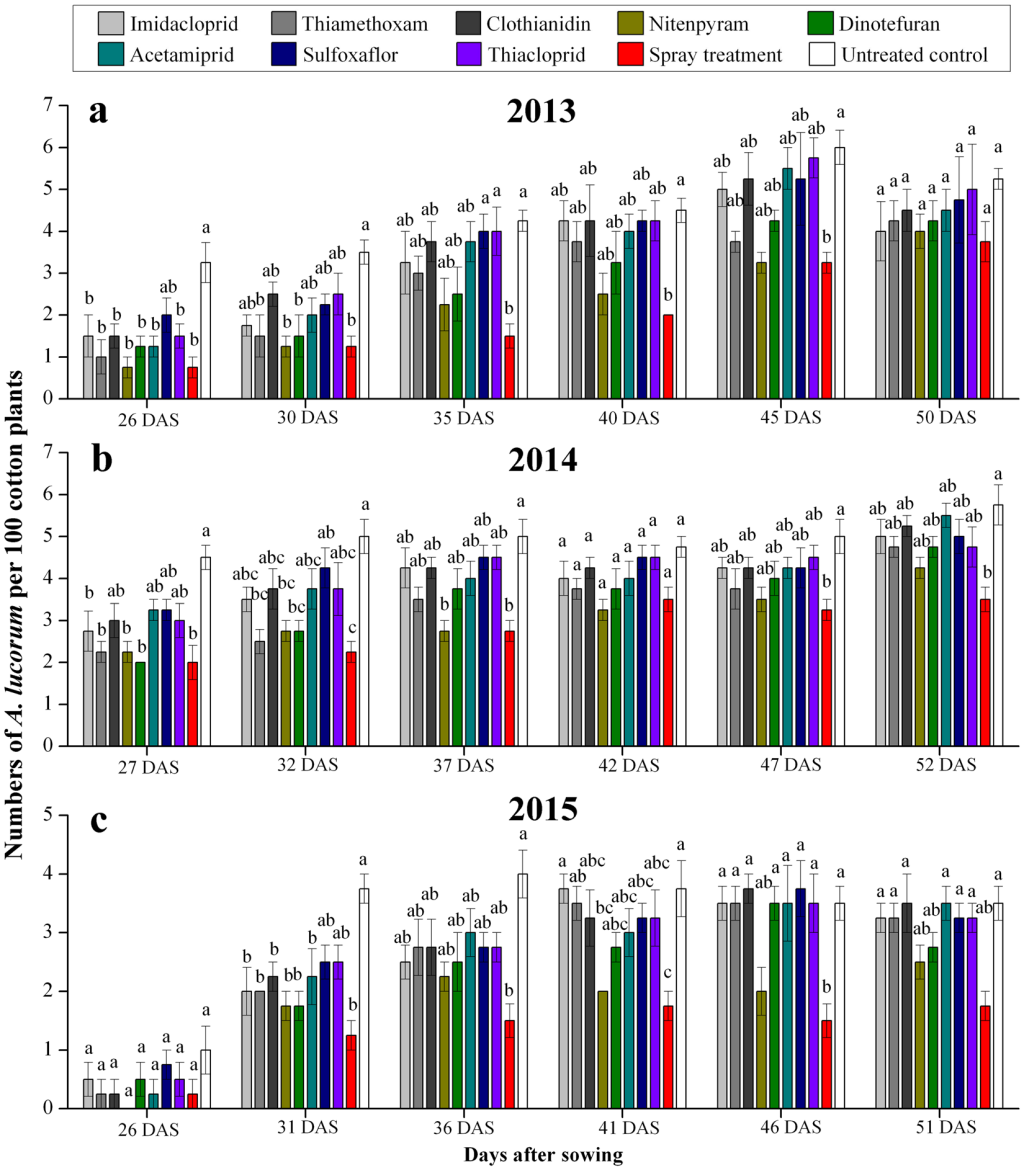
Population dynamics (**a**–**c**) of *Apolygus lucorum* per 100 plants in each plot in 2013, 2014 and 2015. Different letters indicate significant differences among the treatments (Tukey’s HSD test, *P* < 0.05). DAS = days after sowing.

At 26 days after sowing (DAS), in 2013, the number of *A. lucorum* in plots treated with neonicotinoid seed treatments and spray treatments was significantly less than that in the control treatment (*F*_9,39_ = 4.345, *P* = 0.001), except for plots treated with sulfoxaflor. The number of *A. lucorum* in plots treated with nitenpyram, dinotefuran, and thiamethoxam was not significantly different than that in plots with spray treatments but was significantly lower than that in the control treatment at 30 DAS (*F*_9,39_ = 3.750, *P* = 0.003). Among all the neonicotinoid seed treatments, the population densities of *A. lucorum* in the nitenpyram-treated plots was low but not obviously significantly lower than that in the untreated control at 35, 40, 45 DAS, and 50 DAS (35 DAS: *F*_9,39_ = 3.068, *P* = 0.010; 40 DAS: *F*_9,39_ = 2.831, *P* = 0.015; 45 DAS: *F*_9,39_ = 3.899, *P* = 0.002; 50 DAS: *F*_9,39_ = 0.538, *P* = 0.835) (Fig. 1a). In summary, in 2013, the densities of *A. lucorum* were significantly lower in the nitenpyram-treated plots than those in the control plots (*F* = 1.755, *P* = 0.101) (Table 2).

**Table 2:** The number of *Apolygus lucorum* in various neonicotinoid-treated field plots.

Treatment	Year		
	2013	2014	2015
Imidacloprid	3.29 ± 0.58ab	3.96 ± 0.31abc	2.58 ± 0.49ab
Thiamethoxam	2.88 ± 0.54ab	3.42 ± 0.37bc	2.54 ± 0.51ab
Clothianidin	3.63 ± 0.57ab	4.13 ± 0.30abc	2.63 ± 0.52ab
Nitenpyram	2.33 ± 0.49b	3.13 ± 0.29bc	1.75 ± 0.37b
Dinotefuran	2.83 ± 0.53ab	3.50 ± 0.40bc	2.29 ± 0.43ab
Acetamiprid	3.50 ± 0.65ab	4.13 ± 0.31abc	2.58 ± 0.50ab
Sulfoxaflor	3.75 ± 0.54ab	4.29 ± 0.24ab	2.71 ± 0.43ab
Thiacloprid	3.83 ± 0.64ab	4.17 ± 0.27abc	2.63 ± 0.45ab
Spray treatment	2.08 ± 0.48b	2.88 ± 0.26c	1.33 ± 0.23b
Untreated control	4.46 ± 0.43a	5.00 ± 0.17a	3.25 ± 0.46a
*Df*	9, 59	9, 59	9, 59
*F*	1.755	4.441	1.428
*P*	0.101	<0.001	0.202

Values within columns represent the mean numbers of *Apolygus lucorum* per 100 cotton plants per sampling date in each plot.

Different letters indicate significant differences among the different treatments (Tukey’s HSD test, *P* < 0.05).

At 27 and 32 DAS in 2014, the number of *A. lucorum* in plots treated with nitenpyram, dinotefuran, and thiamethoxam was not significantly different than that in plots with the spray treatments but was significantly lower than that in the control treatment (27 DAS: F_9,39_ = 5.497, *P* < 0.001; 32 DAS: *F*_9,39_ = 4.593, *P* = 0.001). At 37 DAS, the *A. lucorum* density in the nitenpyram-treated plots was significantly lower than that in the untreated control plots (*F*_9,39_ = 4.525, *P* = 0.001). At 42, 47, and 52 DAS, there were no significant differences in the *A. lucorum* population densities between the eight neonicotinoid seed treatments and the untreated control (42 DAS: *F*_9,39_ = 2.150, *P* = 0.056; 47 DAS: *F*_9,39_ = 2.092, *P* = 0.063; 52 DAS: *F*_9,39_ = 3.356, *P* = 0.006) (Fig. 1b). In 2014, the densities of *A. lucorum* in the plots treated with nitenpyram, thiamethoxam and dinotefuran were significantly lower than the densities in the untreated plots (*F* = 4.441, *P* < 0.001) (Table 2).

At 31 DAS in 2015, the number of *A. lucorum* in plots treated with the neonicotinoid seed treatment and spray treatment was significantly less than that in the control treatment (*F*_9,39_ = 5.048, *P < *0.001), except for plots treated with sulfoxaflor and thiacloprid. At 41 DAS, the *A. lucorum* density in the nitenpyram-treated plots was significantly lower than that in the untreated control plots but not significantly different than that in the other neonicotinoid treatments and spray treatments (*F*_9,39_ = 2.791, *P* = 0.017) (Fig. 1c). In 2015, the densities of *A. lucorum* were significantly lower in the nitenpyram-treated plots than those in the control plots (*F* = 1.428, *P* = 0.202) (Table 2).

During the 2013, 2014 and 2015 field experimental periods, the neonicotinoid seed treatment and sampling date significantly affected the numbers of *A. lucorum* (*P* < 0.05). The interaction between the neonicotinoid seed treatment and the sampling date had no significant effect on the numbers of *A. lucorum* in any of the years (Table 3).

**Table 3:** The effects of the neonicotinoid seed treatments, sampling date, and interactions on the numbers of *Apolygus lucorum*.

Source	*df*	2013	*P-*values	2014	*P-*values	2015	*P-*values
		*F*		*F*		*F*	
Treatment	9	13.084	**<0.001**	37.905	**<0.001**	100.020	**<0.001**
Sampling date	5	69.713	**<0.001**	19.345	**<0.001**	16.068	**<0.001**
Treatment × sampling date	45	0.540	0.992	0.628	0.966	1.069	0.370

Bolded *P-*values indicate significant treatment effects (*P* < 0.05).

### Effect of neonicotinoid seed treatments on the percentage of damaged plants

**Figure 2:**
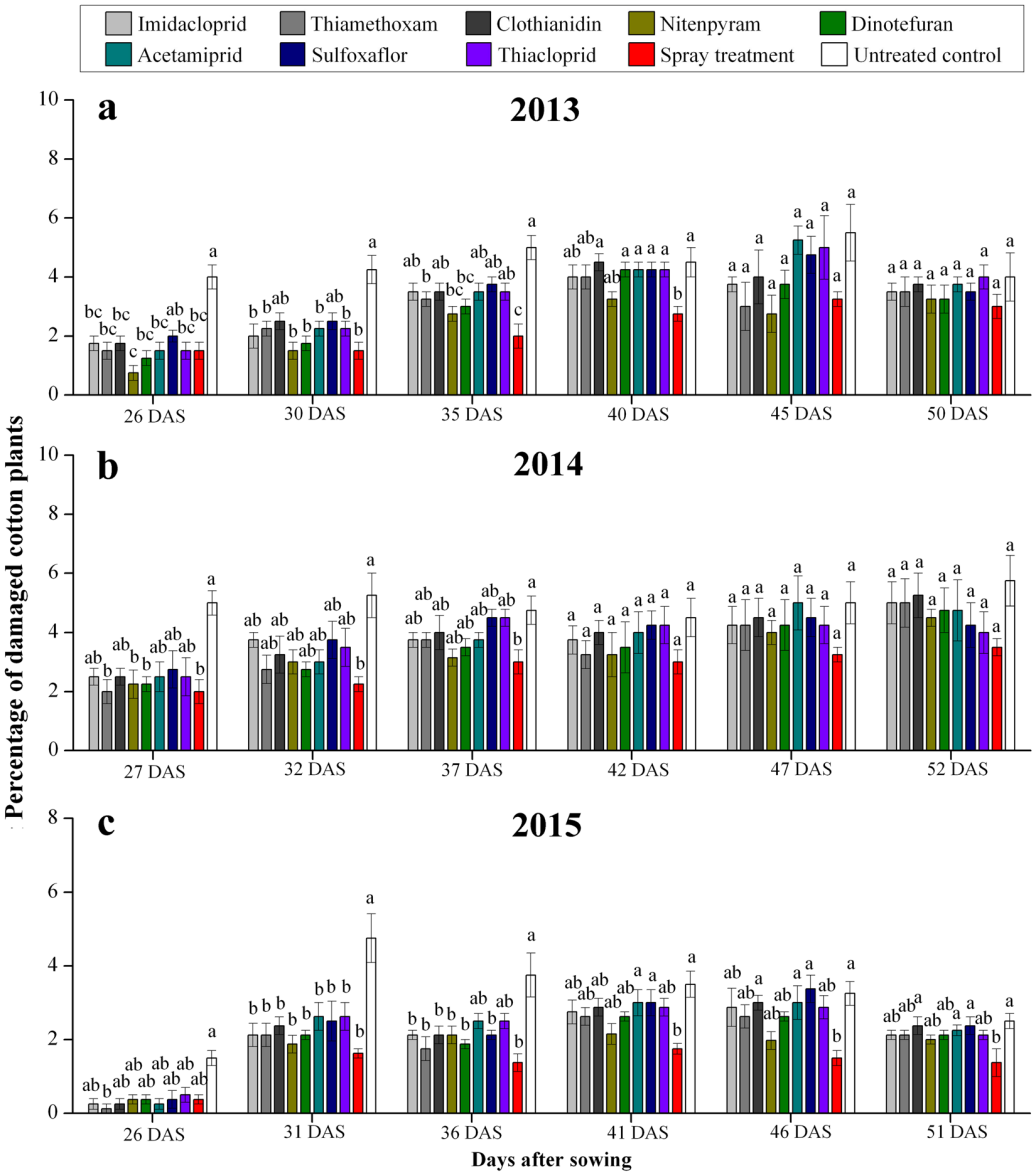
Percentage of damaged cotton plants (**a**–**c**) in each plot at each sampling date in 2013, 2014 and 2015. Different letters indicate significant differences among the treatments (Tukey’s HSD test, *P* < 0.05). DAS = days after sowing.

*A. lucorum* causes growing seedlings to wither, cotton leaves to break and multi-headed seedlings. At 26 and 30 DAS in 2013, the percentage of damaged plants by *A. lucorum* in plots treated with the neonicotinoid seed treatment and spray treatment was significantly less than that in the control treatment (26 DAS: *F*_9,39_ = 5.929, *P* < 0.001; 30 DAS: *F*_9,39_ = 5.281, *P* < 0.001), except for plots treated with sulfoxaflor and clothianidin. The percentage of damaged plants by *A. lucorum* in plots treated with nitenpyram and dinotefuran was not significantly different than that with the spray treatments but was significantly lower than that in the control treatment at 30 DAS (*F*_9,39_ = 6.516, *P* < 0.001). At 40, 45, and 50 DAS, there were no significant differences in the percentage of damaged plants by *A. lucorum* between the eight neonicotinoid seed treatments and the untreated control (40 DAS: *F*_9,39_ = 2.895, *P* = 0.014; 45 DAS: *F*_9,39_ = 1.923, *P* = 0.087; 50 DAS: *F*_9,39_ = 0.507, *P* = 0.857) (Fig. 2a). In 2013, the percentage of damaged plants by *A. lucorum* in the nitenpyram-treated plots was significantly lower than that in the untreated plots (*F* = 1.868, *P* = 0.079) (Table 4).

**Table 4:** Percentage of damaged cotton plants in various neonicotinoid-treated field plots.

Treatment	Year		
	2013	2014	2015
Imidacloprid	3.08 ± 0.96ab	3.83 ± 0.82ab	2.05 ± 0.94a
Thiamethoxam	2.92 ± 0.90 ab	3.50 ± 1.07 ab	1.90 ± 0.93a
Clothianidin	3.33 ± 1.02 ab	3.92 ± 0.96 ab	2.17 ± 1.00a
Nitenpyram	2.46 ± 1.12b	3.42 ± 0.79 ab	1.92 ± 0.82a
Dinotefuran	2.88 ± 1.16 ab	3.50 ± 0.92 ab	1.96 ± 0.83a
Acetamiprid	3.42 ± 1.36 ab	3.83 ± 0.97 ab	2.27 ± 1.03a
Sulfoxaflor	3.46 ± 1.04 ab	4.00 ± 0.67 ab	2.30 ± 1.04a
Thiacloprid	3.42 ± 1.31 ab	3.83 ± 0.74 ab	2.25 ± 0.90a
Spray treatment	2.33 ± 0.77 b	2.83 ± 0.58b	1.34 ± 0.49a
Untreated control	4.54 ± 0.60a	5.04 ± 0.43a	3.21 ± 1.11a
*Df*	9, 59	9, 59	9, 59
*F*	1.868	2.607	0.799
*P*	0.079	0.015	0.619

Values within columns represent the mean percentage of damaged cotton plants per sampling date in each plot.

Different letters indicate significant differences among the different treatments (Tukey’s HSD test, *P* < 0.05).

In 2014, the percentage of damaged plants by *A. lucorum* in plots treated with nitenpyram, dinotefuran and thiamethoxam was significantly lower than that in the control treatment at 27 DAS (*F*_9,39_ = 2.638, *P* = 0.022) (Fig. 2b). The percentage of damaged plants by *A. lucorum* in the neonicotinoid-treated plots and spray treatments was lower than that in the control treatment, but the effects were not statistically significant (*F* = 2.607, *P* = 0.015) (Table 4).

At 26 DAS in 2015, the percentage of damaged plants by *A. lucorum* in plots treated with thiamethoxam was significantly lower than that in the control treatment (*F*_9,39_ = 2.128, *P* = 0.059). At 31 DAS, the percentage of damaged plants by *A. lucorum* in the neonicotinoid treatments and spray treatments was significantly lower than that in the control treatment (*F*_9,39_ = 4.939, *P* < 0.001). At 36 DAS, the percentage of damaged plants by *A. lucorum* in the neonicotinoid treatments, except for acetamiprid and thiacloprid, was significantly lower than that in the control treatment (*F*_9,39_ = 5.086, *P* < 0.001) (Fig. 2c). There were no significant differences in the percentage of damaged plants by *A. lucorum* between the eight neonicotinoid seed treatments and the untreated control (*F* = 0.799, *P* = 0.619) (Table 4).

The neonicotinoid seed treatments and sampling date significantly affected the percentage of damaged plants by *A. lucorum* (*P* < 0.05). However, the interaction between the neonicotinoid seed treatments and the sampling date did not significantly affect the percentage of damaged plants (Table 5).

**Table 5:** The effects of the neonicotinoid seed treatments, sampling date, and interactions on the percentage of damaged cotton plants.

Source	*df*	2013	*P-*values	2014	*P-*values	2015	*P-*values
		*F*		*F*		*F*	
Treatment	9	13.346	**<0.001**	17.210	**<0.001**	12.457	**<0.001**
Sampling date	5	60.173	**<0.001**	53.387	**<0.001**	144.945	**<0.001**
Treatment × sampling date	45	1.252	0.154	1.399	0.998	1.213	0.189

Bolded *P*-values indicate significant treatment effects (*P* < 0.05).

## Discussion

Neonicotinoids have a high efficacy against a broad spectrum of sucking pests and have a high degree of versatility^14^. During the seedling stage of cotton, most individuals in the *A. lucorum* population are at the nymph stage and have no flight capability. In addition, *A. lucorum* do not frequently move in cotton fields with stable environmental conditions and an adequate food supply^20^. Neonicotinoid seed treatments can allow for a more precise targeting of the active ingredient on which *A. lucorum* feed in cotton plants. The results of the field trials over three years confirmed that among all the neonicotinoid seed treatments, the nitenpyram seed treatments at the rate of 4 g AI kg^−1^ had the greatest efficacy in controlling *A. lucorum* during the seedling stage of cotton. The effect of the nitenpyram seed treatments was similar to that of spraying insecticide. Nitenpyram, which is a second-generation neonicotinoid, has a broad-spectrum efficacy and a systemic and translaminar action, and the importance of this neonicotinoid is increasing rapidly in the Chinese market^21^. Zhang *et al*.^22^ also demonstrated that the granular treatments of nitenpyram at sowing can reduce *A. lucorum* and *Aphis gossypii* (Glover) infestations during the seedling to blooming stages in Bt cotton. Currently, imidacloprid is most commonly applied as seed dressings to control sucking pests throughout the cotton seedling stage in China. However, Zhang *et al*.^23^ showed that the imidacloprid seed treatment had a relatively poor control efficacy against *A. lucorum*. Our study also showed that the nitenpyram seed treatment had a higher control efficiency against *A. lucorum* than imidacloprid. Therefore, nitenpyram used as a seed treatment can manage early season *A. lucorum* and provide effective protection in Bt cotton fields.

The differences in the control efficacy of the neonicotinoids against *A. lucorum* might be related to the residues of the neonicotinoids in the cotton leaves. There were higher residues of nitenpyram in the cotton leaves, which corresponded to the higher efficacies against *A. lucorum* ^24^. The differences in the residue levels of the neonicotinoids in the cotton leaves were likely related to the water solubility of the insecticides. Neonicotinoids are translocated to cotton leaves through xylem tissues^25^. Nitenpyram has a higher water solubility (590 g/L) than the other neonicotinoids and should be more readily available for uptake^26^. In addition, the differences in the control efficacies of the neonicotinoids may be related to the toxicities of the insecticides on this pest. Nitenpyram exhibited a high toxicity against *A. lucorum* ^27^ and other piercing-sucking pests, such as *Aphis gossypii* ^21^ and *Bemisia tabaci* ^28^. The efficacy of nitenpyram against *A. lucorum* decreased during the later sampling periods. This decreased efficacy can be attributed to a very low concentration of the neonicotinoids in the cotton leaves, which is due to the dissipation of the neonicotinoids.

The neonicotinoid seed treatments did not affect the emergence rate of cotton seeds in the laboratory. The seedling emergence in the field did not differ between the neonicotinoid-treated and untreated control plots. Moreover, the neonicotinoid seed treatments obviously increased the plant heights of the cotton seedlings, which may enhance the ability of the plants to protect against exogenous disturbances. Although the neonicotinoid seed treatments had little effect in controlling *A. lucorum* after mid-Jun, all treatments were needed for spraying insecticides to control *A. lucorum* during the bud stage and the flowering and boll-opening stages. Finally, no significant differences were found in the cotton yields between the neonicotinoid seed treatments and the spray treatment. The neonicotinoid seed treatments reduced the frequency of the pesticide application by at least 3 applications in this study while reducing the growing labor costs.

Insecticides have a direct contact toxicity against natural enemies in cotton fields by spraying and are more threatening to natural enemies than seed treatments^29^. Currently, farmers in China spray insecticides primarily based on the degree of damage caused by *A. lucorum* in their cotton fields and rarely consider the role of natural enemies in controlling pests^30^. The neonicotinoid seed treatments could reduce the number of insecticide sprays throughout the seedling stage of cotton, indicating a lower risk to beneficial arthropods. Ladybeetles, syrphidae, spiders and aphid parasitoids are the predominant natural enemy species in Chinese cotton fields^31^. Nitenpyram had no obvious impact on the population fecundity and growth of ladybeetles^32^. Therefore, the nitenpyram seed treatment may be relatively safe for these natural enemies. The extensive use of insecticides, such as imidacloprid, has a negative impact on the health of honey bees and other pollinators^33, 34^. The neonicotinoid seed treatments could reduce the overall spraying of imidacloprid throughout the seedling stage of cotton, enhancing the conservation of the pollinating insects, e.g., honey bees. In addition, nitenpyram was relatively safer for honey bees than imidacloprid. The oral toxicity of nitenpyram to honey bees (LD50 = 0.138 µg/bee) was lower than that of imidacloprid (LD50 = 0.0037 µg/bee)^26^.

Neonicotinoid seed treatments with moderate persistence and water solubility have raised concerns regarding environmental contamination^35^. Applications of neonicotinoids as seed treatments result in approximately 1.6% to 20% of the active ingredient being absorbed by the target crop^36^. Therefore, the bulk of the active ingredients enter the soil^37^. The concentrations of neonicotinoids in soils after their application typically decline rapidly due to plant uptake, degradation leaching, and absorption^38^. However, neonicotinoids may persist under some conditions, and successive applications of neonicotinoids may result in residue accumulation in the soil^37^. For example, imidacloprid residues in winter barley fields in the United Kingdom plateaued after 6 successive annual applications^37^. Residues of neonicotinoid insecticides after 3–4 successive annual applications tend to plateau to a mean concentration of less than 6 ng/g in agricultural soils in Southwestern Ontario, Canada^39^. Therefore, the wide spread application of nitenpyram as a seed treatment for the control of *A. lucorum* in the future warrants further investigations regarding the persistence of this compound with a high leaching potential in soils in a typical field crop ecosystem dominated by cotton production.

In the current study, we demonstrated that seed treatments with nitenpyram at the rate of 4 g AI kg^−1^ can protect cotton plants from *A. lucorum* infestations throughout the seedling stage. To date, seed treatment with fungicides has already been widely used for the control of seedling diseases in cotton, such as *Rhizoctonia solani* Kuhn and *Verticillium dahliae* Kleb^40^. Therefore, seed treatments combining nitenpyram with fungicides should be a suitable choice for controlling piercing-sucking pests, such as *A. lucorum*, and diseases during the seedling stage of cotton.

## Materials and Methods

### Cotton seeds and insecticides

The transgenic Bt cotton seeds variety Xinqiu-1 were supplied by Shandong Xinqiu Agricultural Science and Technology Co., Ltd. The seeds were delinted and selected before the insecticide seed treatments. The following eight neonicotinoid insecticides were used in this study: imidacloprid (Gaucho 600 g L^−1^ FS; Bayer CropScience (China) Co., Ltd, Hangzhou, China), thiamethoxam (Cruiser 70% WS, Syngenta Crop Protection (Suzhou) Co., Ltd, Suzhou, China), clothianidin (Poncho 600 g L^−1^ FS, Bayer CropScience LP, Monheim, Germany), nitenpyram (50% SG, Jiangshan Agrochemical & Chemical Co., Ltd, Nantong, China), dinotefuran (20% SG, Mitsui Chemicals, Inc., Bangkok, Thailand), acetamiprid (20% SG, Shandong United Pesticide Industry Co., Ltd, Tai’an, China), Sulfoxaflor (22% SC, Dow AgroSciences LLC., Shanghai, China), and thiacloprid (48% SC Noposion Agrochemicals Co., Ltd, Shenzhen, China). These formulations were diluted to a uniform slurry with water before the seed treatment. The seeds were treated by applying a slurry of the insecticide via a syringe to 3 kg lots of cotton seeds in plastic bags. The bags were inflated and shaken by hand for 1 min until uniform coverage was achieved; the seed was allowed to dry before planting.

### Effect of neonicotinoid seed treatments on seed germination

The seed germination under different neonicotinoid seed treatment conditions was assessed in glass Petri dishes (15 cm in diameter and 3 cm in height) in the laboratory. The trials included eight neonicotinoid seed treatments at the rate of 4 g AI kg^−1^ seed and an untreated control. Silica sand and the Petri dishes were washed with water and dried at 120 °C for 24 h. The Petri dish was filled with moistened sand, and the moisture content was maintained at 60% by spraying water daily. Then, 50 plump seeds were pushed into the sand at a 1 cm depth in each dish and covered by moistened sand. Each treatment was conducted with six replications, and 2 dishes were established in each replication. These dishes were placed randomly on shelves in a germination chamber set at 25 ± 1 °C, 65 ± 2% RH and a photoperiod of 12:12 (L:D) h. The number of germinated seeds was counted 7 days after sowing.

### Field experiments

In the 2013, 2014, and 2015 cotton growing seasons, field experiments were performed to evaluate the efficacy of eight neonicotinoid seed treatments in the management of *A. lucorum* at a cotton breeding base of Shandong Xinqiu Agricultural Science and Technology Co., Ltdin Xiajin, Shandong, China (site: 36.93°N, 115.95°E). The soil type was silty loam (clay 12.15%, silty 61.88%, sandy 25.97%), pH = 7.53 and the organic content was 1.41%. Farmers usually sow cotton seeds in late April, cover the seeds with a translucent plastic film to maintain warmth and harvest in mid-September.

The ten treatments included eight neonicotinoid seed treatments at the rate of 4 g AI kg^−1^ seed (i.e., imidacloprid, thiamethoxam, clothianidin, nitenpyram, dinotefuran, acetamiprid, sulfoxaflor, and thiacloprid), an untreated treatment, and a foliar spraying treatment and were arranged in a randomized complete block design with four replications. The foliar spray treatment was performed in accordance with the pest management program of the cotton breeding base. The details of the spraying treatment are shown in Table S1. The cotton seeds were sowed on April 25, 2013, April 23, 2014, and April 27, 2015. The seed furrows were established using a mechanical furrow opener and were 80 cm apart and 5 cm in depth. The seeds were sowed via manual dibbling at three seeds per hole and a 25 cm distance. Approximately 20 kg seeds per ha were used, and the plant densities were approximately 45,000 per ha. Each plot consisted of ten rows 7 m long containing approximately 260 cotton plants. The plots were separated by 1.6 m of bare cultivated ground. The pendimethalin was applied at the rate of 800 g AI ha^−1^ after sowing, and then, each row was covered with a translucent plastic film.

The emergence condition was assessed by counting the number of cotton seedlings in each plot 15 days after sowing on May 10, 2013, May 8, 2014, and May 12, 2015. The number of *A. lucorum* was counted every five days six times in 100 randomly selected plants in each plot beginning on May 21, 2013, May 20, 2014, and May 19, 2015, and continuing until mid-June. The number of *A. lucorum* in each plot was determined using the knock-down method^41, 42^. This method consisted of pulling parts of the cotton plants over a rectangular white-colored pan (60 × 35 × 3 cm); then, the plant material was immediately shaken, and the number of dislodged *A. lucorum* adults and nymphs was counted. *Apolygus lucorum* causes growing seedlings to wither, cotton leaves to break and many-headed seedlings^43^. The number of cotton plants exhibiting *A. lucorum* damage was counted in 100 randomly selected plants per plot, and the percentage of damaged plants was assessed.

### Data analysis

All statistical analyses were performed using SPSS statistical software (version 18.0, SPSS Inc., Chicago, IL, USA). Statistically significant mean values were compared using a one-way ANOVA, followed by the Tukey’s HSD method (*P* < 0.05). Significant differences in the *A. lucorum* population density and percentage of damaged plants by *A. lucorum* in various neonicotinoid-treated field plots vs. untreated control plots were determined using repeated-measures analysis of variance, with treatments as the factors and the sample date as the split-plot factor. The percentage of damaged plants was transformed using the arcsine-square root prior to the analysis, but untransformed data are presented.

## Electronic supplementary material

Supplementary Information Table S1
