# Supplementary material for: Nitenpyram seed treatment effectively controls against the mirid bug Apolygus lucorum in cotton seedlings
Source: Sci Rep. 2017 Aug 17;7:8573. doi: 10.1038/s41598-017-09251-9 (PMC5561033; doi:10.1038/s41598-017-09251-9)
Supplement: Supplementary file 1 — Supplementary Information Table S1 [file 41598_2017_9251_MOESM1_ESM.doc]

**Supplementary Information:**

**Nitenpyram seed treatment effectively controls against the mirid bug *Apolygus lucorum* in cotton seedlings**

Zhengqun Zhang^b^#, Yao Wang^a^#, Yunhe Zhao^a^, Beixing Li^a^, Jin Lin^a^, Xuefeng Zhang^a^, Feng Liu^a^, and Wei Mu^a^*

a. *College of Plant Protection, Shandong Agricultural University, 61 Daizong Street, Tai’an 271018, China*

b*.* *College of Horticulture Science and Engineering, Shandong Agricultural University, 61 Daizong Street, Tai’an 271018, China*

#These authors contributed equally to this work.

**Corresponding authors:**

^*^Prof. Wei Mu, College of Plant Protection, Shandong Agricultural University, 61 Daizong Street, Tai’an, Shandong 271018, P.R. China. Tel: +86-538-8242611, Email: [muwei@sdau.edu.cn](mailto:muwei@sdau.edu.cn)

**Table S1.** The information of insecticides applied by foliar spray in cotton fields in 2013, 2014 and 2015.

| Date |  | Spray areas | Formulations and dosage |
| --- | --- | --- | --- |
| 2013 | 12 May | Spray treatment | 5% imidacloprid EC, 450 mL hm^-1^ |
|  | 19 May | Spray treatment | 5% imidacloprid EC, 450 mL hm^-1^; 25 g L^-1^ lambda-cyhalothrin EC, 900 mL hm^-1^ |
|  | 28 May | Spray treatment | 5% acetamiprid EC, 300 mL hm^-1^; 25% pymetrozine WP, 240 g hm^-1^ |
|  | 4 June | Spray treatment | 5% acetamiprid EC, 300 mL hm^-1^; 25 g L^-1^ lambda-cyhalothrin EC, 900 mL hm^-1^ |
| 2014 | 15 May | Spray treatment | 25g L^-1^ lambda-cyhalothrin EC, 900 mL hm^-1^ |
|  | 22 May | Spray treatment | 70% imidacloprid WG, 30 g hm^-1^; 25% pymetrozine WP, 240 g hm^-1^ |
|  | 30 May | Spray treatment | 70% imidacloprid WG, 30 g hm^-1^; 25g L^-1^ lambda-cyhalothrin EC, 900 mL hm^-1^ |
| 2015 | 13 May | Spray treatment | 48% chlorpyrifos ME, 450 mL hm^-1^ |
|  | 20 May | Spray treatment | 5% imidacloprid EC, 450 mL hm^-1^; 200g L^-1^ carbosulfan EC, 300 mL hm^-1^ |
|  | 27 May | Spray treatment | 5% imidacloprid EC, 450 mL hm^-1^; 1.8% abamectin EC, 450 mL hm^-1^ |
|  | 2 June | Spray treatment | 5% imidacloprid EC, 450 mL hm^-1^; 25g L^-1^ lambda-cyhalothrin EC, 900 mL hm^-1^ |

EC, SC, WP, WG, ME and AS in columns represent the codes of pesticide formulations. EC represents emulsifiable concentrate; SC represents aqueous suspension; WP represents wettable powder; ME represents micro-emulsion; AS represents aqueous solution.
